# Snail Mucus Filtrate Reduces Inflammation in Canine Progenitor Epidermal Keratinocytes (CPEK)

**DOI:** 10.3390/ani12141848

**Published:** 2022-07-21

**Authors:** Laura Messina, Fabio Bruno, Patrizia Licata, Davide Di Paola, Gianluca Franco, Ylenia Marino, Alessio Filippo Peritore, Salvatore Cuzzocrea, Enrico Gugliandolo, Rosalia Crupi

**Affiliations:** 1 Department of Veterinary Science, University of Messina, 98168 Messina, Italy; laura.messina@studenti.unime.it (L.M.); fabio.bruno@studenti.unime.it (F.B.); plicata@unime.it (P.L.); rcrupi@unime.it (R.C.); 2 Department of Chemical, Biological, Pharmaceutical and Environmental Science, University of Messina, 98166 Messina, Italy; davide.dipaola@unime.it (D.D.P.); gianluca.franco@studenti.unime.it (G.F.); ylenia.marino@studenti.unime.it (Y.M.); alessiofilippo.peritore@unime.it (A.F.P.); salvator@unime.it (S.C.)

**Keywords:** canine atopic dermatitis, *Helix aspersa* Muller, snail secretion filtrate

## Abstract

**Simple Summary:**

Canine atopic dermatitis (cAD) is a clinical syndrome characterized by inflammatory and allergic manifestations. Recent studies have demonstrated that cAD has many common characteristics with human AD and this assertion is derived from the assumption that domestic dogs share the environment with their owners. Several therapeutic approaches can be used in the management of cAD; in our research, we used the mucus secreted by *Helix aspersa* Muller. To clarify the development of cAD, we employed cell lines of canine epidermal keratinocytes (CPEK). Our results highlight the anti-inflammatory capacity of mucus in reducing the inflammatory process produced during cAD.

**Abstract:**

Atopic dermatitis (AD) is an inflammatory and allergic disease, whose multifactorial etiopathogenesis is the consequence of the link between the genetic, immunological and environmental components. The complexity and difficulty in understanding the causes that trigger or exacerbate this pathology makes it difficult, once diagnosed, to proceed with a targeted and effective therapeutic process. Today, the new frontiers of research look to natural and innovative treatments to counteract the different manifestations of dermatitis. From this point of view, the mucus secreted by *Helix aspersa* Muller has proven, since ancient times, to be able to neutralize skin diseases. To study canine atopic dermatitis (cAD), we used cell lines of canine epidermal keratinocytes (CPEK) that are optimal to understand the biological reactivity of keratinocytes in vitro. The data obtained from our study demonstrate the anti-inflammatory capacity of snail secretion filtrate (SSF) in counteracting the production of proinflammatory cytokines produced during cAD, highlighting the opportunities for further studies to be able to identify new, natural and safe treatments for cAD and to open new frontiers for veterinarians and owners.

## 1. Introduction

The International Committee for Allergic Diseases of Animals (ICADA) classified canine atopic dermatitis (cAD) as a genetically predisposed inflammatory and pruritic allergic skin disease with many possible manifestations; several clinical features are associated with IgE antibodies that are most frequently directed against environmental allergens [1,2]. cAD is subjective by the interaction of both, genetic and environmental factors, by abnormalities of the immune system, by its interaction with the nervous system and by defects in the epidermal barrier. It is becoming increasingly clear that cAD is a clinical syndrome and not a single disease. Recently, it was demonstrated that cAD has several common characteristics with human dermatitis and this claim stems from the assumption that, particularly domestic dogs, share the environment with their owners [1,3]. Much progress has been made in recent years in terms of understanding the role of skin in identifying new treatments [4]. In particular, dogs reflect the complexity of human disease; in fact, in these animals, atopic dermatitis has become more common since the animals have acquired the same lifestyles as the owners, for example increased exposure to clean indoor environments and increased consumption of processed foods. All of these new behaviors have contributed to increasing the risk of developing allergic diseases [5,6]. Given the characteristics of the disease, the impact on quality of life of both dogs and owners is evident, in terms of impact on health, well-being and the human-animal bond [7,8]. As in humans, hallmarks of cAD are an abnormal immune response to environmental allergens and an impaired epidermal barrier [9]. The enormous interest in the study of cAD is also due to the great potential of the dog as a model for the human species. In fact, it has been ascertained that there are many aspects that can be compared regarding the two species and the knowledge obtained in the field of veterinary medicine has greatly contributed to expanding the knowledge about human atopic dermatitis. Even in humans, this disease causes enormous inconveniences and is considered, in its most serious forms, one of the main disabling diseases. Usually, the development of the disease involves a first phase in which there is sensitization to environmental allergens located in a space, for example in the home, since both dust and mites penetrate the skin and lead to both recruitment and stimulation of inflammatory cells; inflammatory mediators, such as cytokines and chemokines, play a key role in the development of the disease. In Western societies, contact with various allergens is almost constant and continuous; however, a reality in which an increasingly excessive use of antibiotics, pesticides, disinfectants and very frequent baths favors a reduction in contact with microorganisms and this explains how this pathology has a very rapid and incisive evolution in recent decades [10,11]. The correct diagnosis is made by excluding other itchy diseases that have overlapping clinical signs [12]. The therapeutic option to be used is strictly determined by the patient’s condition and the severity of the disease. Generally, the management of cAD needs a multimodal therapeutic approach which involves the following: allergen-specific immunotherapy, antipruritic drugs (Janus kinase inhibitors, calcineurin inhibitors) and barrier restructuring agents (nutraceuticals and herbal medicines) [13,14] but also skin and coat hygiene and care. Today, it has become increasingly urgent to identify new therapeutic treatments that can be used for the management of cAD and a key role is played by substances of animal origin. Under the great umbrella of natural substances, our attention was focused on snail slime. Previous studies, conducted in our laboratories, have shown that snail slime, secreted by the snail *Helix aspersa* Muller, has strong anti-inflammatory activity, demonstrated in in vivo studies [15,16]. The idea of using mucus or snail secretion has its roots in the history of humans, when in ancient times, the mucus was used for the management of skin disorders; today, it is proposed as a component for the formulation of parapharmaceutical products for wound management and as a constituent of cosmetic products. Current knowledge about the mucus produced by *Helix aspersa* Muller indicates that mucus is rich in hyaluronic acid, mucopolysaccharides, polyphenols and bioactive minerals [17]. These substances increase the adhesion of mucus to the skin, acting as a protective barrier, while the polyphenols counteract the damage associated with oxidative stress. Moreover, the mucus is characterized by reparative activity [18], due to its emollient, antimicrobial and adhesive properties [19]. Thanks to these characteristics, the mucus extracted from *Helix aspersa* Muller has been successfully used as a re-epithelizing treatment in the management of burn wounds in adult patients [20]. The characteristics of the mucus produced by *Helix aspersa* Muller have attracted our attention; therefore, recently, we have deepened the study of the biological properties of snail secretion filtrate (SSF). The mucus used was achieved after manual stimulation of the snails with a sterile cotton swab tip and then subjected to a series of filtrations to obtain SSF. Based on the knowledge acquired on SSF and on the basis of the study of inflammatory processes, in our research, we have explored the action of SSF in a cell line of canine progenitor epidermal keratinocytes (CPEK) as an alternative treatment in the management of cAD.

## 2. Materials and Methods

CPEK cells were treated with or without SSF in the presence or absence of LPS. A cell viability assay was performed to test SSF toxicity and the protective effect of SSF after LPS stimulation. Additionally, enzyme-linked immunosorbent assay (ELISA) kits were used to detect the levels of IL-6, IL-8 and IL-17A and the level expression of mRNA for COX1, COX2 and TNF-alfa. 

### 2.1. Snail Secretion Filtrate

*Helix aspersa* Muller mucus was kindly delivered by Snail S.R.L.S (Messina, Italy). The integrated system of ground farming of 100% Italian Helix Aspersa snails provides a careful choice of selected foods to enhance the intrinsic properties of the slime. The snails live in the open air, immersed in a suitable vegetative environment and specially designed to suit their needs and requirements. The breeding was cruelty-free. In particular, the mucus was obtained mechanically by manually stimulating snails with a sterile cotton swab tip. The extraction cycle was not affected by the use of chemical agents or mechanical stimulations. In the first step, the mucus was filtered with a coarse filter to stabilize the pH, after which the mucus was distributed through a filtration series of 3 different filters (10 microns, 1 micron, 0.22 micron; Pall) and then stored at 4 °C. In particular, the use of the 0.22 micron filter is important to eliminate impurities and endotoxins [16]. No animals were purchased for experimental purposes and no ethical committee permission was mandatory.

### 2.2. Cell Culture and Treatment

As previously described [21], the proliferative canine keratinocyte cell line CPEK (96 CELLnTEC, Zen-Bio Inc., Durham, NC, USA) was used in the present study. Cells were resuspended in CnT-09 media (96 CELLnTEC, Zen-Bio Inc.), counted and plated 2 × 10^5^ on sterile 96- or 12-well cell culture plates. CPEK cells were stimulated with 1 mL of 20 ug/mL LPS (Sigma-Aldrich, Saint Louis, MO, USA) in presence or not of SSF at different concentrations, and the plates were incubated for an additional 24 h at 37 °C before harvesting. Fifth to 10th-passage CPEK cells were employed in these experiments, and all assays were executed in triplicate.

### 2.3. MTT Assay

To evaluate the viability of cells, The cells were resuspended in CnT-09 media (96 CELLnTEC, Zen-Bio Inc.), counted and plated 2 × 10^5^ on sterile 96 well cell culture plates and exposed to different concentrations of SSF for 24 h. The MTT 3-(4,5-dimethylthiazol-2-yl)-2,5-diphenyltetrazolium bromide) assays were performed using the CyQUANT™ MTT Cell Viability Assay (Invitrogen, ThermoFisher), according to the manufacturer’s protocols. 

### 2.4. Measurement of Cytokine Production

Cells were resuspended in CnT-09 media (96 CELLnTEC, Zen-Bio Inc.), counted and plated 2 × 10^5^ on sterile 12-well cell culture plates. CPEK cells were stimulated with 1 mL of 20 ug/mL LPS (Sigma-Aldrich) in presence or not of SSF at different concentrations, and the plates were incubated for an additional 24 h at 37 °C before harvesting. Supernatants collected for each experimental condition were used for cytokine measurement using specific canine ELISA kits for IL-6, IL-8 and IL-17A (DuoSet ELISA Kit R&D System, DY1609; DY1608; DY5848).

### 2.5. RT-PCR

The cell pellets collected for each experimental group were used for RNA isolation using the RNeasy Mini Kit (Qiagen, Milan, Italy) according to manufacturer protocols. RNA was quantified using a Nanodrop spectrometer, and subsequently an equivalent quantity of RNA for each sample used for cDNA synthesis using iScriptTM cDNA Synthesis Kit (Bio-Rad, Milano, Italy) according to manufacturer protocols. Real-time PCR analysis was performed by the SYBR Green method using the QuantiTect Primer Assay (Qiagen) for Cf_GAPDHS_1_SG, Cf_PTGS2_1_SG, Cf_PTGS1_1_SG Cf_TNFAIP1_1_SG, according to manufacturer protocols. Real-time PCR was performed using a Bio-Rad CFX Real-Time PCR ((Bio-Rad, Milano, Italy) Detection System. Fold change in mRNA level was determined using the −∆∆Ct data analysis method [22].

### 2.6. Statistical Analysis

The data were analyzed by one-way ANOVA followed by Tukey’s test for multiple comparisons. A *p*-value of less than 0.05 was considered significant. Data are representative of at least three independent experiments. °°° *p* < 0.001 versus CTR; * *p* < 0.05 versus LPS; *** *p* < 0.001 versus LPS.

## 3. Results

### 3.1. Effect of SSF on CPEK Cell Viability 

**Figure 1:**
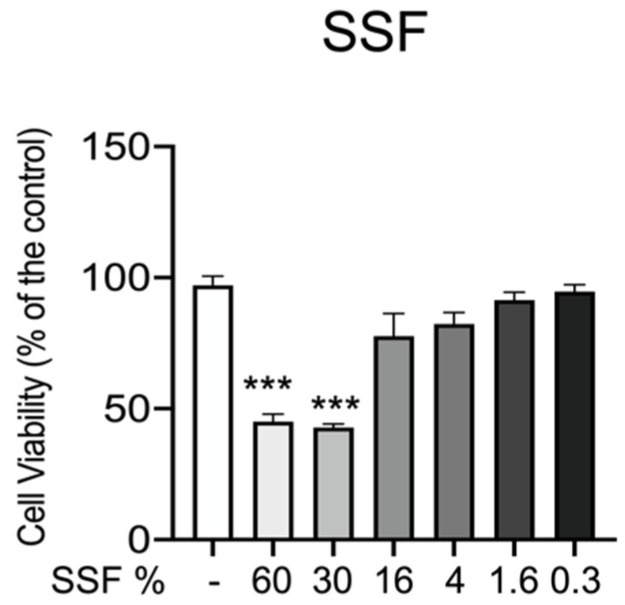
Role of SSF on CPEK viability. Cell viability was assessed by MTT tetrazolium dye. Concentration of 60% and 30% significantly decreased cell viability. Data representative of at least three experiments, means ± SEM *** *p* < 0.001 versus control.

In the first step, we performed an MTT assay to know whether SSF aggravates a toxic effect on CPEK cell viability. As reported in Figure 1, CPEK cells were incubated with different concentrations of SSF (from 60% to 0.3%) for 24 h. Our data showed that at concentrations of 60% and 30%, SSF significantly reduced cell viability. On the other hand, SSF at concentrations of 16% to 0.3% does not significantly alter cell viability. 

### 3.2. Effect of SSF on IL-6, IL-8 and IL-17A Release

**Figure 2:**
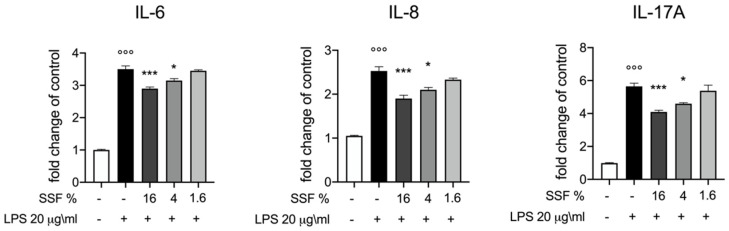
Effect of SSF on IL-6, IL-8 and IL-17A expression. ELISA quantification of IL-6, IL 8, IL-17A after LPS intoxication and lSSF treatment. SSF was efficient at notably reducing IL-6, IL 8, IL-17A. Data representative of at least three experiments,°°° *p* < 0.001 versus CTR; * *p* < 0.05 versus LPS; *** *p* < 0.001 versus LPS.

As displayed in Figure 2, we investigated the influence of SSF on the release of IL-6, IL-8 and IL-17A by CPEH cells. Our results reported that the concentrations of 16% and 4% significantly inhibited the release of all cytokines.

### 3.3. Effect of SSF on COX1, COX2 and TNF-α Expression

**Figure 3:**
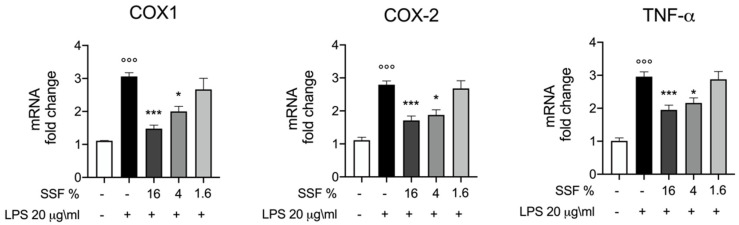
Protective effect of SSF in LPS-induced intoxication in CPEK cells: mRNA levels of COX1, COX2 and TNF-alfa one hour post LPS stimulation and SSF treatment. Data representative of at least three experiments,°°° *p* < 0.001 versus CTR; * *p* < 0.05 versus LPS; *** *p* < 0.001 versus LPS.

To better evaluate the anti-inflammatory effect of SSF in LPS-induced inflammation, we evaluated the mRNA expression level of the COX1, COX2 and TNF-α as key mediators of the inflammatory process. In Figure 3, we reported that SSF is able to decrease significantly the mRNA expression levels for COX1, COX2 and TNF-alfa, in particular at 16% and 4%. 

## 4. Discussion

The aim of our research was to investigate the effect of SSF in an in vitro model of canine dermatitis. Atopic dermatitis is a clinical syndrome that affects both people and animals [23,24]. In the treatment of cAD, there are four key points that determine the choice of treatment and concern, which are as follows: time, inflammation, itching and the appearance of infections [25]. To these, one can also add the chronicity and the severity of the lesions that determine the choice of drugs, also considering the effectiveness, side effects and costs [26]. Often, cAD and its relapses are treated with antimicrobial and antipruritic drugs that can exacerbate factors such as dysbiosis or intestinal permeability, thus favoring the development of antibiotic-resistant microbes [27]. It was reported, in fact, that intestinal permeability is a frequently employed marker for several intestinal health, as increased intestinal permeability may lead to inflammation caused by bacterial components, such as lipopolysaccharide (LPS), that are able to cross the epithelial barrier [28]. In this context, the new frontiers of research are identifying new therapeutic approaches to minimize the use of broad-spectrum therapies, such as glucocorticoids or cyclosporins, in favor of approaches that are more targeted, natural and safe for both owners and animals. In our study, we identified and analyzed a pool of inflammatory markers in an in vitro model of cAD, such as L-6, IL-8, IL-17A, and TNF-alfa, because the cytokine environment plays a relevant role in both skin morphology and innate immunity, and keratinocytes are players in innate immunity. Notably, the transcriptional regulation of many of these markers was modulated by nutraceutical exposure [29] because it involves binding of toll-like receptors and downregulation of the predominately Th2-mediated allergic response [30]. It is now accepted that the regular utilization of food supplements (polyphenols, essential fatty acids, vitamins or probiotics) has beneficial effects on animals [31]. Epidemiological data regarding the development of cAD and the analysis of the impact on patients’ lives today justify the need to identify new and sustainable therapeutic strategies aimed at counteracting both the development and complications of atopic dermatitis. In this context, the use of snail slime is placed as a therapeutic option, used in an applicative manner since ancient times in traditional medicine. Today, detailed studies on its chemical composition and biological characterization are still lacking. The use of snail slime in the cosmetic field is now recognized for the following properties that distinguish it: emollient, lubricant and protective. In addition to the aforementioned peculiarities and characteristics, snail slime is also characterized by its antimicrobial [32] and healing effects [15,33]. There are several findings that demonstrate the absence of cytotoxicity in different cell lines, such as in human keratinocytes (HaCaT), human dermal fibroblasts (MRC-5) and murine embryo fibroblasts (NIH-3T3) [17]. The regenerative properties are particularly related to the amount of both allantoin and glycolic acid but recently, it has been shown that the mucus in toto has a bigger effect than that of the distinct molecules [34]. Based on these data, it is likely that there is a synergy linked to the action of several molecules present in the mucus; in addition to this, there is also the ratio of the various components, which represents a key factor in determining its biological activity. Based on previous data obtained on snail slime, in this study, we used it to modulate the expression of inflammatory mediators, such as cytokines, proposing it as a new therapeutic approach for the treatment of cAD. Our starting point was the evaluation of the toxicity of SSF on CPEK cells; from the analysis of the data, it emerged that SSF is toxic at concentrations between 60% and 30%, probably due to the high concentration of active compounds at this dose. Subsequently, the cells were stimulated with LPS and the SSF showed an important protective effect on cell viability (16%, 4%, 1,6%). Our findings revealed that SSF is able to significantly diminish the release of IL-6, IL-8 and IL-17A. IL-6 is a proinflammatory cytokine expressed in the acute phase of inflammation and plays a role in the increase and exacerbation of Th2-mediated diseases [35]. IL-8 stimulates the activation and enrolment of innate immune cells, such as neutrophils, at the site of inflammation [36]. Moreover, IL-8 triggers cells by stimulating exocytosis and degranulation of storage proteins, particularly linked to the process of wound healing and inflammation [37,38]. IL-8 stimulates the proliferation, growth and viability of vascular endothelial cells, which are involved in angiogenesis [39]. IL-17 stimulates the production of antimicrobial factors by increasing the host’s defenses and it also produces pro-inflammatory cytokines by stimulating the recruitment of neutrophils and macrophages to the site of infection [40]. IL-17 expression increases in acute lesions in AD skin compared to uninvolved skin in humans [41,42]; mice studies showed that the deficiency of IL-17A mitigates the development of skin inflammation [43]. During chronic lesions in the skin of atopic subjects, the expression of IL-17 appears relatively low compared to IL-22, as observed in human medicine [44]. The data obtained in our experiments show that SSF is able to act as an antiallergic agent by inhibiting the expression of TNF-α. TNF-α is a recognized proinflammatory cytokine; its inhibition is now studied as a valid approach to prevent allergic phenomena [45]. Published data show that the over expression of Th2 cytokines is prevalent in the acute phase of lesions caused by atopic dermatitis [46], while in the meantime, other proinflammatory cytokines, such as IL-6 and TNF-α, are over expressed in the chronic phase. [46]. SSF treatment was able to reduce TNF-α mRNA expressions. The COX enzymes COX-1 and COX-2 play a predominant role in allergic reactions and mast cell-mediated inflammation [47]. Both inflammatory cytokines and endotoxins stimulate the production of COXs; therefore, lowering the level of expression, in particular of COX-2, can be considered an attractive strategy for the development of antiallergic drugs [48]. SSF exhibited substantial effects in destroying the level of protein expression, involving TNF-α and COX-2. Therefore, SSF might be considered as an anti-allergic approach in the management of cAD. 

## 5. Conclusions

In conclusion, our study demonstrated the efficacy of SSF to counteract the inflammatory process associated with cAD in the canine keratinocyte cell line. Today, we have several drug options available for the management of cAD but the identification of natural and safe treatments for cAD opens new frontiers for both veterinarians and owners. The therapy of cAD must be implemented through a multimodal approach, aimed at giving the patient a better degree of comfort through the reduction or disappearance of itching and secondary manifestations of the disease. Recently, great attention has been focused on all therapies aimed at providing skin integrity and promoting the restoration of the skin barrier. This therapeutic line can be accompanied by an immune-modulating therapy, aimed at normalizing an unbalanced immune response that favors the onset of the disease. In the last decade, the study and identification of unconventional therapies, such as natural substances, have been intensified to reduce, complement or supplant drug therapies for the management of cAD. Data collected from this study refer to a possible use of SSF in the management of the cAD, but in the future, it will be necessary to better investigate the mechanism of action of SSF. 

## Data Availability

Data sharing is not applicable to this article.
